# Effects of Telerehabilitation on Quality of Life, Physical Capacity, and Symptom Burden Within the Post–COVID-19 Condition: Systematic Review and Meta-Analysis of Randomized Controlled Trials

**DOI:** 10.2196/95368

**Published:** 2026-10-27

**Authors:** Xiaojing Huang, Yidan Tang, Dongsen Liu, Ryan Donald Burns, Wonwoo Byun

**Affiliations:** 1 Department of Health & Kinesiology College of Health University of Utah Salt Lake City, UT United States; 2 Yunnan Provincial Institute of Sports Science Kunming, Yunnan China; 3 Department of Sports Rehabilitation School of Sports Medicine and Rehabilitation Beijing Sport University Beijing China

**Keywords:** telerehabilitation, post–COVID-19 condition, health-related quality of life, digital rehabilitation, meta-analysis

## Abstract

**Background:**

Post–COVID-19 condition (PCC) is characterized by persistent symptoms and functional limitations that impair quality of life, and rehabilitation is an important component of its management. Because access to conventional face-to-face rehabilitation may be limited, telerehabilitation has emerged as an accessible alternative. However, the effectiveness of telerehabilitation across clinical outcomes remains uncertain, and the influence of different delivery modes is not well understood.

**Objective:**

This study aimed to evaluate the effects of telerehabilitation on health-related quality of life (HRQoL), physical capacity, and symptom burden in adults with PCC, and to determine whether delivery mode (synchronous, asynchronous, or hybrid) modified these effects.

**Methods:**

Seven databases (PubMed, Cochrane Library, Web of Science, Scopus, Embase, EBSCO, and PEDro) were searched for randomized controlled trials published from January 2020 through December 2025. Eligible trials enrolled adults with PCC and compared telerehabilitation with usual care or another control condition. The primary outcome was HRQoL; secondary outcomes included functional capacity assessed by the 6-minute walk test and sit-to-stand test, dyspnea, fatigue, and symptoms of anxiety and depression. Risk of bias was assessed using the Cochrane Risk of Bias 2 tool. Mean differences or standardized mean differences (SMDs) with 95% CIs were pooled using random-effects models with the Hartung-Knapp-Sidik-Jonkman adjustment. Heterogeneity was quantified using I² and τ², with 95% prediction intervals reported where estimable. Certainty of evidence was evaluated using the GRADE (Grading of Recommendations Assessment, Development and Evaluation) approach.

**Results:**

A total of 22 randomized controlled trials involving 2294 participants were included. Telerehabilitation was associated with significant improvements in HRQoL (SMD 1.26, 95% CI 0.07-2.45; *P*=.04), dyspnea (SMD 1.95, 95% CI 0.60-3.31; *P*=.005), and functional aerobic capacity (6-minute walk test : mean difference 77.79 meters, 95% CI 30.44-125.14; *P*=.001). Fatigue was also significantly reduced (SMD 0.89, 95% CI 0.16-1.62; *P*=.02), but no significant effects were observed for lower-limb strength (sit-to-stand test: SMD 0.55, 95% CI –0.15 to 1.25; *P*=.12) or symptoms of anxiety and depression (SMD 0.12, 95% CI –0.13 to 0.38; *P*=.36). No significant differences between delivery modes were observed for HRQoL (*P*=.93), functional aerobic capacity (*P*=.29), or dyspnea (*P*=.16). A significant subgroup difference was observed for lower-limb strength (*P*=.02).

**Conclusions:**

Telerehabilitation may improve quality of life, aerobic capacity, and symptom burden in PCC and offer an accessible option for post-COVID rehabilitation, although the certainty of evidence was generally limited. Asynchronous delivery appears feasible for general reconditioning, whereas its benefit for lower-limb strength and the value of synchronous supervision remain uncertain.

**Trial Registration:**

PROSPERO CRD42023490863; https://www.crd.york.ac.uk/PROSPERO/view/CRD42023490863

## Introduction

Post–COVID-19 condition (PCC), also referred to as long COVID, is a common long-term consequence of SARS-CoV-2 infection. The World Health Organization defines it as symptoms occurring about 3 months after probable or confirmed COVID-19. These symptoms last for at least 2 months and cannot be explained by another diagnosis [1,2]. Common manifestations include fatigue, dyspnea, postexertional symptoms, reduced exercise capacity, cognitive difficulties, and psychological symptoms. These problems often coexist and can lead to sustained limitations in daily functioning, participation, and health-related quality of life (HRQoL) [3-6]. Although the acute phase of the pandemic has passed, PCC remains common, and its prolonged course continues to create substantial health care needs for patients and health systems [2,7,8].

Physical rehabilitation, particularly pulmonary and exercise training, is widely recognized as a central component of PCC management [9,10]. Studies have demonstrated that structured rehabilitation can significantly improve exercise capacity and alleviate dyspnea in convalescent patients [11-14]. However, access to conventional face-to-face rehabilitation remains limited due to health care resource constraints, geographic barriers, and workforce shortages [15]. These challenges underscore the need for alternative rehabilitation delivery models that are both effective and scalable within existing health care systems.

Telerehabilitation, defined as the delivery of rehabilitation services through information and communication technologies, has emerged as a promising approach to address limited access to conventional rehabilitation in individuals with PCC [16,17]. By reducing travel requirements and allowing treatment to be completed at home, telerehabilitation may support continuity of care, scheduling flexibility, and access for patients who are unable to attend regular facility-based programs [18,19]. However, remote delivery also introduces important challenges. Participation and treatment response may be influenced by access to technology, digital literacy, platform usability, patient engagement, and the availability of timely clinical feedback. Existing digital health studies in PCC have also reported considerable variation in intervention design and limited reporting of implementation and safety outcomes [20,21].

Recent studies generally support the beneficial effects of telerehabilitation on physical function and symptom management in populations with PCC [22-24]. However, interpretation of these findings remains limited by differences in patient characteristics, intervention components, outcome instruments, and follow-up duration. Earlier reviews were also based largely on small or early trials and frequently combined different forms of remote rehabilitation within a single treatment category [23,25-27]. A recent updated meta-analysis expanded the evidence base to 28 randomized controlled trials (RCTs) and reported improvements in physical function and several symptom outcomes, but its primary quantitative synthesis remained focused on physical and symptom-based outcomes [28]. Consequently, uncertainty remains regarding the overall effect of telerehabilitation on HRQoL and the extent to which treatment effects vary across different digital delivery modes.

A further gap concerns the assessment of patient-centered outcomes. Because patients with PCC experience multiple and fluctuating symptoms, the overall impact of the condition cannot be adequately represented by a single objective test. HRQoL captures how symptoms and functional limitations affect patients’ daily lives and was therefore selected as the primary outcome of this review. This emphasis is consistent with rehabilitation frameworks that prioritize functioning and participation [29,30] and with PCC core outcome initiatives calling for standardized, patient-centered assessment [31,32]. Objective measures of aerobic capacity and lower-limb function, including the 6-minute walk test (6MWT) [33] and sit-to-stand test (STS) [34,35], were included as complementary secondary outcomes.

The mode of telerehabilitation delivery represents another important but unresolved issue. Synchronous programs provide real-time supervision, immediate feedback, and opportunities to adjust treatment during a session, but they require greater clinician time and scheduling coordination. Asynchronous programs offer greater flexibility and may reduce resource demands, but they provide less immediate feedback and rely more heavily on patient self-management. Hybrid programs combine elements of both approaches, although their added value has not been clearly established [35,36]. Existing reviews have often pooled these delivery modes or examined telerehabilitation as a single category, making it difficult to determine whether the amount and timing of supervision influence treatment effects. Evidence comparing delivery approaches remains limited, and previous analyses have not consistently examined synchronous, asynchronous, and hybrid interventions across HRQoL, functional, and symptom outcomes [37,38].

This systematic review and meta-analysis evaluated the effects of telerehabilitation on HRQoL as the primary outcome and on functional capacity, dyspnea, fatigue, and symptoms of anxiety and depression as secondary outcomes in adults with PCC, compared with usual care or another control. We further examined whether intervention effects differed across synchronous, asynchronous, and hybrid delivery modes. By synthesizing HRQoL across validated instruments and assessing delivery mode within a single analytical framework, this review provides an updated, patient-centered synthesis of the available randomized evidence.

## Methods

### Design and Search Strategy

The research adhered to the PRISMA (Preferred Reporting Items for Systematic Reviews and Meta-Analyses) reporting guideline and was registered in PROSPERO (CRD42023490863) (Multimedia Appendix 1) [39]. A comprehensive electronic literature search was conducted from January 2020 to December 2025 in several databases including PubMed, the Cochrane Library, Web of Science, PEDro, Scopus, Embase, and EBSCO. Search terms and synonyms included adults with PCC or long COVID, along with Telerehabilitation, or Remote Rehabilitation, or Digital Rehabilitation, tailored to each search engine (Multimedia Appendix 2). We manually reviewed the reference lists of included studies and pertinent review to find additional papers not included. Study eligibility criteria were defined according to the PICOS (Population, Intervention, Comparison, Outcome, and Study design) framework (Table 1) [40].

**Table 1:** PICOS (Population, Intervention, Comparison, Outcome, and Study design) model.

Domain	Description
Population	Adults with post–COVID-19 condition or long COVID.
Intervention	Telerehabilitation interventions delivered remotely through digital or telecommunication technologies, including programs conducted in home-based or nonclinical settings.
Comparison	Contrast with usual care, no intervention group or placebo.
Outcomes	Reported data on health-related quality of life, physical capacity, symptom burden (fatigue and dyspnea), or psychological outcomes assessed using validated scales.
Study design	Randomized controlled design.

### Study Selection and Data Extraction

We examined only RCTs with full text that compared telerehabilitation therapies with usual care, placebo, or control in adults (older than 18 years) with PCC. Telerehabilitation was defined as rehabilitation delivered remotely through digital or telecommunication technologies, allowing patients to perform rehabilitation in home-based or nonclinical settings, with guidance, feedback, monitoring, or education provided by health professionals or structured digital systems and without in-person contact [16]. Home-based programs that lacked ongoing remote supervision or interactive support were not classified as telerehabilitation. Based on the mode of delivery, interventions were classified into 3 categories: synchronous, delivered in real time with live, therapist-supervised interaction (eg, videoconferencing or live online sessions); asynchronous, delivered without real-time interaction through self-paced tools (eg, mobile apps, web platforms, prerecorded modules, or remote monitoring); and hybrid, combining real-time and self-paced components within the same program [41]. The delivery mode of each included study is shown in Table 2. We did not impose any limitations regarding comorbidities or medication usage during the rehabilitation procedure. Excluded were studies without sufficient statistical data for extraction, reviews, case reports, observational studies, and any other type of publications (eg, guidelines, protocols, comments, conference abstracts, and editorials). Two reviewers (XH and YT) conducted independent searches of papers in each database. Disagreements were resolved by consensus or by involving a third reviewer. We collected information on authors, publication year, participants, intervention details, and outcome data.

**Table 2:** Characteristics of included telerehabilitation interventions.

Study	Telerehabilitation intervention	Telerehabilitation protocol	Delivery mode	Outcome	Follow-up
Alsharidah et al [42], 2023	Supervised pulmonary telerehabilitation including breathing exercises, aerobic training, and resistance training delivered via live videoconferencing	6 weeks; 3 sessions per week, 60-80 minutes per session, intensity guided by ACSM^a^ recommendations	Synchronous	6-minute walk test, pulmonary function, and health-related quality of life	Week 6
Bileviciute-Ljungar et al [43,44], 2024-2025	Group-based multidisciplinary telerehabilitation including physiotherapy, exercise therapy, psychoeducation (pain, sleep, and cardiopulmonary symptoms), and psychological and occupational therapy delivered online	8 weeks; group sessions 2 hours per day, 3 days per week; weekly 20-minute individual sessions; additional self-exercise ≥3 hours per week using ExorLive app	Hybrid	Health-related quality of life (SF-36^b^), pain intensity, pain-related ICF^c^ categories, anxiety and depression	6 months
Calvo-Paniagua et al [45], 2024	A multicomponent program delivered via videoconferencing, including sanitary and health education, respiratory therapy and breathing exercises, aerobic exercise and physical conditioning, active mobilization, motor control, and balance exercises, and occupational therapy focused on activities of daily living	7 weeks, 18 sessions on alternate days, 40 minutes per session	Synchronous	Modified Borg Scale, mMRC^d^ scale, SGRQ^e^, 6MWT^f^	1 month and 3 months
Del Corral et al [46], 2023	Home-based respiratory muscle training using a threshold pressure device and remote supervision	8 weeks, 40 minutes per day, 6 times per week	Synchronous	EQ-5D-5L, Ruffier test, 1-minute STS^g^, Anxiety, Depression	NR^h^
Demir et al [47], 2026	A comprehensive exercise-based telerehabilitation program including breathing and respiratory control exercises, inspiratory muscle training using incentive spirometry, upper- and lower-limb strengthening with elastic resistance bands, balance training, and walking exercises	6 weeks, 3 sessions per week, 60 minutes per session, progressive exercise intensity over time	Synchronous and asynchronous	6MWT, mMRC, Chalder Fatigue Scale	Week 12
Elyazed et al [48], 2024	Home-based pulmonary rehabilitation including walking training, diaphragmatic breathing exercises, and resistance exercises for upper and lower limbs	12 weeks; walking 30-60 minutes, 5 days per week; breathing exercises 10-15 minutes twice daily; low-load resistance training	Asynchronous	SF-36, 6MWT, Fatigue	Week 12
de Araújo Furtado et al [49], 2023	Multicomponent functional telerehabilitation training including aerobic, strength, agility, balance, speed, and power exercises delivered remotely with real-time supervision	8 weeks; 3 sessions per week; 45-60 min per session; Borg RPE^i^ 6-8; progressive multicomponent training + remote health education lectures	Synchronous	6MWT, 30-second STS, flex-arm, get up and go); handgrip strength; depression (Beck Depression Inventory); anxiety (BAI^j^)	Week 8
King et al [50], 2023	Walking training, upper- and lower-limb strengthening and combination aerobic movements	10 weeks, twice weekly, at least 16 sessions	Synchronous	SGRQ, 6MWT, 5STS, fatigue	NR
Kerling et al [51], 2024	Online-supported individualized physical activity program including aerobic endurance, strength, recovery, breathing, and flexibility exercises with wearable-based monitoring	3 months; target ≥150 minutes per week moderate-intensity activity (60-75% HR_max_^k^); individualized plan; weekly remote consultations (weeks 1, 4, and 8) via phone or video; Garmin activity tracker support	Asynchronous	Exercise capacity (V̇O_2peak_, peak power output); fatigue (FAS^l^); health-related quality of life (SF-36); anxiety and depression (HADS^m^); work ability; physical activity	3 months
León-Herrera et al [52], 2024	Multimodal online rehabilitation including physical activity, respiratory exercises, cognitive rehabilitation, diet, sleep hygiene, emotional management, and community resources	8 weeks; 1 session per week; 90 minutes per session; group videoconference via Google Meet + Moodle platform (educational materials, exercises, and forums)	Hybrid	Quality of life (SF-36 physical and mental), physical function (sit-to-stand), cognitive function (MoCA^n^), anxiety or depression (HADS), insomnia (ISI^o^), self-efficacy, health literacy, patient activation	3 months
Longobardi et al [53], 2023	Individualized home-based exercise training including aerobic, resistance, and flexibility exercises with partial live online supervision	16 weeks; 3 sessions per week; 60-80 minutes per session; intensity progressed by RPE	Hybrid	HRQoL^p^ (SF-36), functional capacity (STS), cardiopulmonary fitness, pulmonary function, persistent symptoms	NR
Li et al [54], 2022	Breathing control, thoracic expansion, aerobic exercise, and LMS^q^ exercises remotely monitoring	6 weeks; 3-4 sessions per week, 3 levels: 30%-60% HR^r^	Asynchronous	Quality of life, 6MWT, dyspnea, FVC^s^	NR
McNarry et al [55], 2022	Tele-education, using a handheld inspiratory flow resistive device, remote adherence monitoring	8 weeks; 3 sessions per week, 20 minutes each session, >80% SMIP^t^, 6 set × 6 rep	Asynchronous	Quality of life, dyspnea index, maximal inspiratory pressure, V̇O_2max_	NR
McGregor et al [56], 2024	Live online supervised group exercise plus psychological support sessions delivered remotely	8 weeks; weekly live group exercise sessions and 6 psychological support sessions; home-based, equipment-free	Synchronous	Health-related quality of life (PROMIS-PROPr^u^), fatigue, depression, pain interference, physical function, mental health outcomes	3, 6, and 12 months
Okan et al [57], 2022	Respiratory control, pursed lip breathing, diaphragmatic breathing exercises, and telemedicine and supervision	5 weeks; everyday, 3 times per day, 1 set × 10 repetitions	Hybrid	6MWT, FVC, dyspnea, SGRQ^v^, mMRC	NR
Philip et al [58], 2022	Tele-education and mood telemonitoring, teleconsultation, and telecommunication	6 weeks, on-demand and adapted to each participant	Synchronous	Quality of life, dyspnea, anxiety and depression, VAS^w^, GAD-7^x^, CAT^y^	NR
Paneroni et al [59], 2024	Home-based, self-directed aerobic and strengthening exercise program with progressive intensity, supplemented by scheduled teleconsultations and physiotherapist video supervision	4 weeks; 6 sessions per week; approximately 60 minutes per session; 24 total sessions; biweekly physiotherapist video calls; weekly nurse teleconsultations; exercise intensity tailored by SPPB^ad^; and oxygen desaturation	Asynchronous	6MWT, 1-minute-sit-to-stand (1MSTS), FEV_1_^z^, FVC, FEV_1_/FVC, MIP^aa^, FSS^ab^ Fatigue, SF-12^ac^, Barthel Index Dyspnea	2 months
Rodriguez-Blanco et al [60], 2023	10 breathing and strength-based exercises, telematic control via videoconference	14 days, once a day, 12 repetitions per exercise each day for 30 minutes	Synchronous	Fatigue, 6MWT, 30-second STS, Dyspnea-12, The modified Borg Scale of perceived effort	NR
Samper‑Pardo et al [61], 2023	Tele-education: 3 face-to-face sessions Tele-monitoring: symptom and individual need Health recommendations: diet and sleep Recovery exercises: physical exercise, respiratory physiotherapy exercises, and cognitive stimulation exercises Community resources	12 weeks; human-centered design by individual needs	Asynchronous	Quality of life and 30-second STS, cognitive domains (MoCA), HADS^ae^, ISI^af^, IPAQ GSES-12^ag^	3 months
da Silva et al [62], 2025	Individualized cardiopulmonary telerehabilitation based on functional exercises (stepping, stationary walking, sit-to-stand, upper-limb strengthening, and breathing and mobility exercises) delivered via videoconference	8 weeks; ≥3 sessions per week; 30-50 minutes per session; intensity individualized using 6MST^ah^, 2MSWT^ai^, Borg dyspnea scale, HR and SpO_2_; some sessions supervised live by physiotherapist	Hybrid	Functional capacity (6MST, 2MSWT, and 30CST^aj^), estimated V̇O_2peak_, heart rate demand, physical functioning domain of SF-36	NR
Lai et al [63], 2024	Individualized aerobic exercise training guided by cardiopulmonary exercise testing, supported by wearable heart rate monitoring and mobile app	12 weeks; 3 sessions per week; 40 minutes per session; exercise intensity prescribed using heart rate reserve and ACSM guidelines; app-based monitoring and weekly reminders	Asynchronous	Cardiorespiratory fitness (CPET^ak^); physical activity (IPAQ); exercise self-efficacy; sleep quality (PSQI^al^); health-related quality of life (WHOQOL-BREF^am^)	NR
Yasacı et al [64], 2025	Supervised multicomponent telerehabilitation including diaphragmatic and pursed-lip breathing, relaxation, range of motion, lower-limb strengthening, and aerobic walking exercises delivered via videoconferencing	6 weeks; 2 sessions per week; 45 minutes per session; intensity guided by ACSM and RPE 11-13	Synchronous	Dyspnea (mMRC); pain intensity (NPRS^an^); functional capacity (5-times sit-to-stand test); sleep quality (PSQI); anxiety and depression (HADS)	NR

^a^ACSM: American College of Sports Medicine.

^b^SF-36: 36-Item Short Form Health Survey.

^c^ICF: International Classification of Functioning, Disability and Health.

^d^mMRC: modified Medical Research Council Dyspnea Scale.

^e^SGRQ: St George Respiratory Questionnaire.

^f^6MWT: 6-minute walk test.

^g^STS: sit-to-stand test.

^h^NR: not reported.

^i^RPE: rating of perceived exertion.

^j^BAI: Beck Anxiety Inventory.

^k^HR_max_: maximum heart rate.

^l^FAS: Fatigue Assessment Scale.

^m^HADS: Hospital Anxiety and Depression Scale.

^n^MoCA: Montreal Cognitive Assessment.

^o^ISI: Insomnia Severity Index.

^p^HRQoL: health-related quality of life.

^q^LMS: lower-limb muscle strength.

^r^HR: heart rate.

^s^FVC: forced vital capacity.

^t^SMIP: sustained maximal inspiratory pressure.

^u^PROMIS-PROPr: Patient-Reported Outcomes Measurement Information System–Preference score.

^v^SGRQ: St George Respiratory Questionnaire.

^w^VAS: visual analog scale.

^x^GAD-7: 7-item Generalized Anxiety Disorder scale.

^y^CAT: COPD Assessment Test.

^z^FEV_1_: forced expiratory volume in 1 second.

^aa^MIP: maximal inspiratory pressure.

^ab^FSS: Fatigue Severity Scale.

^ac^SF-12: 12-Item Short Form.

^ad^SPPB: Short Physical Performance Battery.

^ae^HADS: Hospital Anxiety and Depression Scale.

^af^ISI: Insomnia Severity Index.

^ag^IPAQ GSES-12: International Physical Activity Questionnaire and 12-item General Self-Efficacy Scale.

^ah^6MST: 6-minute step test.

^ai^2MSWT: 2-minute stationary walk test.

^aj^30CST: 30-second chair stand test.

^ak^CPET: cardiopulmonary exercise testing.

^al^PSQI: Pittsburgh Sleep Quality Index.

^am^WHOQOL-BREF: World Health Organization Quality of Life Brief.

^an^NPRS: numerical pain rating scale.

### Outcome Measures

The primary outcome was HRQoL. Because HRQoL was assessed using multiple instruments, we included generic measures where available, including the 36-Item Short Form Health Survey (SF-36), the 12-Item Short Form, the EQ-5D, the World Health Organization Quality of Life Brief, and the PROMIS Preference score, as well as respiratory disease–specific measures such as the St George Respiratory Questionnaire and the King Brief Interstitial Lung Disease questionnaire. Secondary outcomes included functional aerobic capacity (6MWT), lower-limb function (STS), dyspnea (modified Medical Research Council scale), Dyspnea-12, Transition Dyspnea Index, and Barthel Dyspnea Index, fatigue (Chalder Fatigue Scale, Fatigue Assessment Scale, and Fatigue Severity Scale), and anxiety and depression (Hospital Anxiety and Depression Scale [HADS]). When a study reported more than 1 eligible instrument for the same outcome, we used the study’s primary or prespecified measure and entered only 1 instrument per study to avoid double-counting. If no primary measure was specified, a generic instrument was prioritized over a disease-specific instrument. The instruments used in each study, together with the direction of scoring, are summarized in Multimedia Appendix 3.

### Risk-of-Bias Assessment

Risk of bias was assessed independently by 2 reviewers (XH and YT) using the Cochrane Risk of Bias tool version 2.0 (RoB 2) [65]. The assessment covered 5 specific domains: the randomization process, deviations from intended interventions, missing outcome data, measurement of the outcome, and selection of the reported result. For each domain, the risk of bias was classified as “low risk,” “some concerns,” or “high risk.” An overall risk-of-bias judgment was derived for each study based on these domain-level assessments. Any discrepancies between the 2 reviewers were resolved through discussion or, when necessary, by consulting a third reviewer.

### Statistical Analysis

Meta-analyses were performed using random-effects models to account for expected clinical and methodological heterogeneity across studies. For continuous outcomes, pooled effect estimates with 95% CIs were calculated as mean differences (MDs) when studies used the same measurement scale and as standardized mean differences (SMDs) when different instruments assessed the same construct (eg, HRQoL, dyspnea, and fatigue). For the SF-36, we used the physical component score rather than combining it with the mental component score; where a study reported only the total score, that value was used instead. Before pooling, all effect directions were harmonized so that positive values indicated clinical benefit: instruments on which higher scores reflect worse health were reverse-coded. The scoring direction of each instrument is summarized in Multimedia Appendix 3. Effect sizes were interpreted according to Cohen guidelines, where SMDs of 0.2, 0.5, and 0.8 represent small, medium, and large effects, respectively [66]. In addition to outcome-specific analyses, an overall pooled analysis was conducted to provide a global summary of the effects of telerehabilitation across outcome domains. Between-study heterogeneity was assessed using the *I*² statistic, the between-study variance (τ^2^), and 95% prediction intervals (PIs). *I*² values of approximately 25%, 50%, and 75% were considered to indicate low, moderate, and high heterogeneity, respectively [67]. To obtain more conservative and robust confidence intervals, particularly in analyses including a limited number of studies, the Hartung-Knapp-Sidik-Jonkman (HKSJ) method was applied [68]. To explore potential sources of heterogeneity, we prespecified subgroup analyses by delivery mode (synchronous, asynchronous, or hybrid). For the primary outcome (HRQoL), we additionally performed exploratory subgroup analyses by participant’s age, time since diagnosis, and intervention duration, with differences assessed using the test for subgroup differences. Sensitivity analyses were performed using a leave-one-out approach by sequentially excluding individual studies to assess the stability of pooled estimates (Multimedia Appendix 4 [42,45-50,52-64]). Publication bias was evaluated using funnel plots and Egger regression test when at least 10 studies were available for a given outcome [69]. Statistical significance was defined as a 2-sided *P* value <.05. All statistical analyses were conducted in R statistical software using metafor and meta packages (R Foundation for Statistical Computing).

### Certainty of Evidence

To assess the certainty of evidence, we applied the GRADE (Grading of Recommendations Assessment, Development and Evaluation) [70-75] approach to the main outcomes, including HRQoL, functional capacity (6MWT and STS), dyspnea, fatigue, and symptoms of anxiety and depression. Two reviewers (XH and YT) independently evaluated the certainty of evidence across the domains of risk of bias, inconsistency, indirectness, imprecision, and publication bias, with disagreements resolved through discussion and consensus. The GRADE assessments are summarized in the Multimedia Appendix 5.

## Results

### Study Selection

A total of 8996 records were identified through database searching across PubMed, the Cochrane Library, Web of Science, Scopus, Embase, EBSCO, and PEDro. After removal of 5157 records before screening, 3839 records were screened by title and abstract, of which 3742 were excluded. In total, 97 full-text papers were assessed for eligibility, and 75 studies were excluded based on predefined criteria. Finally, 22 RCTs were included in the systematic review [42-64], with 20 studies contributing to the quantitative synthesis (meta-analysis). The study selection process is shown in Figure 1.

**Figure 1:**
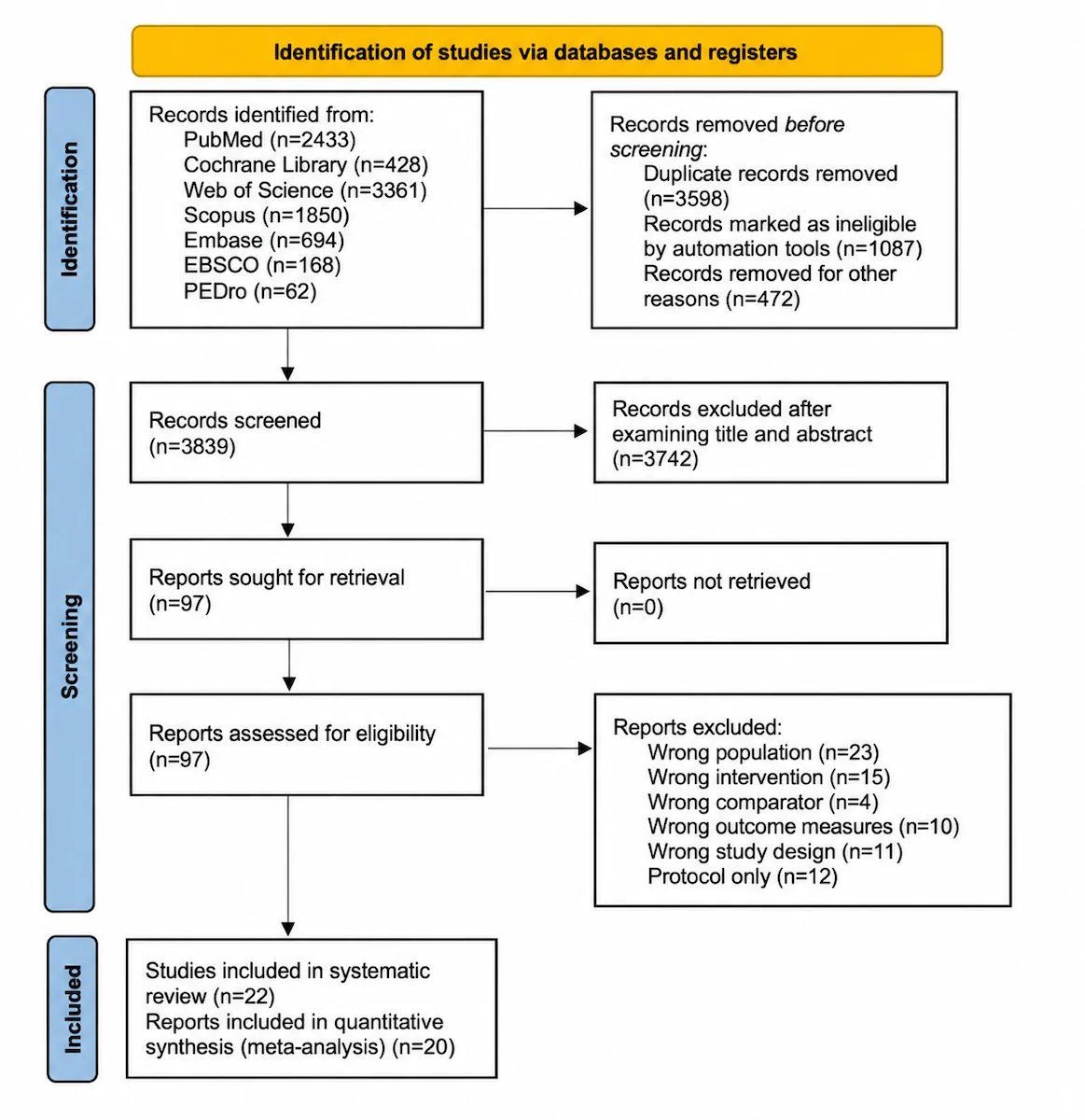
PRISMA (Preferred Reporting Items for Systematic Reviews and Meta-Analyses) flowchart detailing the selection process of randomized controlled trials comparing telerehabilitation with usual care. The search across 7 databases resulted in the inclusion of 22 studies.

### Characteristics of the Included Studies

A total of 22 RCTs involving 2294 participants were included in this meta-analysis. Across studies, participants had mean ages ranging from the mid-40s to mid-50s, and female participants accounted for a higher proportion of the study populations. Sample sizes ranged from 21 to nearly 600 participants. The included trials enrolled clinically heterogeneous populations. Eight trials included patients who had been hospitalized or were recruited after hospital discharge; 3 enrolled community-dwelling, nonhospitalized individuals; and 11 did not clearly report hospitalization status. Acute illness severity ranged from mild to critical: 12 trials included patients with severe or critical illness, 9 enrolled only mild to moderate cases, and 1 did not clearly report severity. Time since infection or hospital discharge ranged from less than 1 month to approximately 24 months, with most studies assessing participants between 3 and 12 months after the acute phase. Sociodemographic and clinical characteristics of the participants across the included studies are summarized in Table 3.

**Table 3:** Sociodemographic characteristics.

Study	Intervention or comparator group	Values, n	Age (years), mean (SD)	Sex, n (%)	Time since diagnosis (months)	Inpatient stay, days	Severity of acute infection
Alsharidah et al [42], 2023	IG^a^: home-based pulmonary telerehabilitation; CG^b^: standardized educational instructions	IG: 24; CG: 24	IG: 23.33 (2.71); CG: 22.58 (2.51)	Female: 100%	4.5	Nonhospitalized	Mild to moderate
Bileviciute-Ljungar et al [43,44], 2024-2025	IG: multidisciplinary group telerehabilitation; CG: waiting list	IG: 67; CG: 42	IG: 43 (9); CG:47 (9)	IG: 15 (22%) males, 52 (78%) females; CG: 5 (12%) males; 37 (88%) females	IG: 11.7 (6.21); CG: 13.4 (7.13)	NR^c^	Mild
Calvo-Paniagua et al [45], 2024	IG: telerehabilitation program based on education, physical exercise, respiratory therapy, and occupational therapy; CG: conventional medical recommendations	IG: 32; CG: 32	IG: 50.8 (8.4); CG: 49.4 (10.0)	IG: 14 (44%) males, 18 (56%) females; CG: 10 (31%) males, 22 (69%) females	14.8 (1.7)	NR	Mild to moderate
Del Corral et al [46], 2023	IMT^d^: inspiratory muscle training IMTsham: sham training RMT^e^: respiratory muscle training RMTsham: sham training	IMT: 22; IMTsham: 22; RMT: 22; RMTsham: 22	IMT: 48.9 (8.3) IMTsham: 45.3 (12.8)RMT: 46.5 (9.6) RMTsham: 45 (10.2)	25 (28%) males; 63 (72%) females	IMT: 11.5 (2.8); IMTsham: 11 (3.5); RMT: 12 (2.2) days; RMTsham: 11.7 (3.1)	NR	Mild to critical
Demir et al [47], 2026	IG: supervised telerehabilitation program； HEG^f^: unsupervised home-based exercise program； CG: patient education only	TRG^g^:12; HEG: 12; CG:12	TRG: 49.50 (12.08); HEG: 38.33 (14.83); CG: 39.50 (17.82)	25 (69%) females; 11 (31%) males	2.3 (1.1)	NR	Mild to moderate
Elyazed et al [48], 2024	IG: home-based pulmonary rehabilitation; CG: usual medical care	IG: 30; CG: 30	56.2 (6.9)	27 (45%) females; 33 (55%) males	≥1 month after acute phase recovery	Nonhospitalized	Mild to moderate
de Araújo Furtado et al [49], 2023	IG: telerehabilitation functional training; CG: no intervention	IG: 16; CG: 16	48.35 (13.0)	18 (56%) females; 14 (44%) males	≥0.7	4.0 (5.3)	Mild to severe
King et al [50], 2023	IG: group telerehabilitation program; CG: usual care	IG:11; CG:10	53 (14)	10 (48%) males; 11 (52%) females	12 (2.2)	IG: 3 (27.3); CG: 1 (10.0)	Mild to critical
Kerling et al [51], 2024	IG: online-supported individualized physical activity intervention; CG: usual lifestyle	IG: 30; CG: 32	46.4 (11.2)	42 (68%) females; 20 (32%) males	≥3	NR	Mild to moderate
León-Herrera et al [52], 2024	IG: online multimodal telerehabilitation; CG: usual care	IG: 67; CG: 67	48.97 (7.64)	113 (84%) females; 21 (16%) males	23.77 (7.87)	NR	NR
Longobardi et al [53], 2023	IG: home-based exercise training; CG: usual care	IG: 25; CG: 25	IG: 60.8 (7.1); CG: 61.2 (7.7)	IG: 12 (48%) males, 13 (52%) females; CG: 13 (52%) males, 12 (48%) females	5.3 (1.2) months since hospital discharge	ICU LOS^h^: 9 (7); hospital LOS: 18 (13) days	Severe to critical
Li et al [54], 2022	IG: unsupervised home-based exercise program; CG: educational instructions	IG:59; CG:60	18-75; 50.61 (10.98)	53 (45%) males; 66 (55%) females	3.4	26.18 (15.25)	Severe to critical
McNarry et al [55], 2022	IG: inspiratory muscle training; CG: usual care	IG: 111; CG: 37	IG: 46.76 (12.03); CG: 46.13 (12.73)	IG: 14% males, 86% females; CG: 5% males, 95% females	9.0 (4.2)	NR	Mild to critical
McGregor et al [56], 2024	IG: online supervised group physical and mental health rehabilitation (REGAIN^i^); CG: best-practice usual care	IG: 298; CG: 287	IG: 56.1 (12.1);CG: 56.2 (12.3)	280 (48%) males; 305 (52%) females	10.6 (4.7) days since hospital discharge	ICU^j^ or HDU^k^ admission: 34% overall	Moderate to critical
Okan et al [57], 2022	IG: breathing exercises; CG: education brochure	IG: 26; CG: 26	IG: 48.85 (10.85); CG: 52.19 (14.84)	27 (52%) males; 25 (48%) females	IG: 2.8 (0.8); CG: 3 (1)	NR	Severe and critical
Philip et al [58], 2022	IG: breathing and well-being program; CG: usual care	IG:74; CG:76	18 years or older IG: 49 (12); CG: 50 (12)	121 (81%) females; 26 (17%) males; 3 (2%) others	IG: 10.9 (4.1); CG: 10.2 (4.3)	NR	Mild to severe
Paneroni et al [59], 2024	IG: self-directed exercise program with biweekly physiotherapist video calls; CG: remote teleconsultation from nursing staff	IG: 40; CG: 39	67.1 (10.3)	57 (72%) males; 22 (28%) females	Recruited at hospital discharge following inpatient rehabilitation	Acute stay: 26.7 (15.4)	Severe
Rodriguez-Blanco et al [60], 2023	IG: respiratory and strength exercises; CG: no intervention, relative rest at home	IG:24; CG:24	IG: 38.75 (15.40); CG:42.58 (11.40)	22 (46%) males; 26 (54%) females	NR	NR	Mild
Samper‑Pardo et al [61], 2023	IG: APP ReCOVery+Usual carel; CG: usual care	IG:52; CG:48	18 years or older 48.28 (9.26)	20 (20%) males； 80 (80%) females	15.9	NR	Mild to critical
da Silva et al [62], 2025	IG: individualized exercise program (functional and accessible exercises) supervised by a physical therapist (videoconference); CG: received guidance on general care and self-monitoring of vital signs (videoconference).	IG: 33; CG: 34	55.0 (11.0)	32 (56%) males; 25 (44%) females	NR	12.0 (9.0)	Severe
Lai et al [63], 2024	IG: telerehabilitation exercise training; CG: physical activity counseling only	IG: 91; CG: 91	39.9 (12.6)	70 (38%) males; 112 (62%) females	3.1	Nonhospitalized	Predominantly mild;
Yasacı et al [64], 2025	IG: supervised telerehabilitation-based exercise program; CG: unsupervised home exercise program	IG: 32; CG: 28	56.0 (12)	31 (52%) males; 29 (48%) females	NR	IG:14.97 (9.22); CG:13.92 (8.25)	Mild to moderate

^a^IG: intervention group.

^b^CG: control group.

^c^NR: not reported.

^d^IMT: inspiratory muscle training.

^e^RMT: inspiratory/expiratory muscle training,

^f^HEG: home exercise group.

^g^TRG: telerehabilitation group.

^h^ICU LOS: intensive care unit length of stay.

^i^REGAIN: Rehabilitation Exercise and Psychological Support After COVID-19 Infection.

^j^ICU: intensive care unit.

^k^HDU: high dependency unit.

Telerehabilitation was delivered synchronously in 9 trials, asynchronously in 7, and through a hybrid approach in 5; 1 additional trial included both synchronous and asynchronous arms. Intervention duration ranged from 2 to 16 weeks, with 2-6 sessions per week. Most programs were multicomponent: aerobic or endurance training was included in 16 trials, breathing exercises or respiratory muscle training in 15, and resistance or strength training in 12, while 5 incorporated psychological or cognitive components. Comparators most commonly included usual care, educational materials, unsupervised exercise, waiting-list controls, or sham interventions. Outcomes were typically assessed at the end of the intervention, and a subset of trials included follow-up at 2, 3, 6, or up to 12 months. Full intervention details are provided in Table 2.

### Risk of Bias and Certainty of the Evidence

All 22 studies were evaluated for bias using the RoB 2 tool (Figure 2), which indicated that 2 of the studies displayed a low risk of bias (9%) [46,56], 17 of them had some concerns (77%) [42,45,47,50-55,57-64], and 3 others had a high risk of bias (14%) [43,44,48,49]. The randomization process (domain 1) was the most methodologically robust domain, with most studies demonstrating a low risk of bias. This indicates that most trials used adequate random sequence generation (eg, computer-generated lists) and allocation concealment mechanisms. In contrast, deviations from the intended interventions (domain 2) and measurement of the outcome (domain 4) presented frequent sources of concern. Due to the nature of telerehabilitation (active exercise training), blinding of participants and personnel was generally not feasible, leading to a rating of “some concerns” or “high risk” in domain 2 for several studies. Similarly, for domain 4, while assessor blinding was implemented in some trials, the use of patient-reported outcome measures, such as quality of life or fatigue, in an unblinded population introduces potential detection bias. Missing outcome data (domain 3) and selection of the reported result (domain 5) were generally rated as low risk, suggesting that most studies performed intention-to-treat analyses and reported outcomes consistent with their protocols. Because these limitations primarily affected the subjective, patient-reported outcomes (HRQoL, dyspnea, and fatigue), the certainty of evidence for these outcomes was downgraded for risk of bias, and their pooled estimates should be interpreted with corresponding caution.

**Figure 2:**
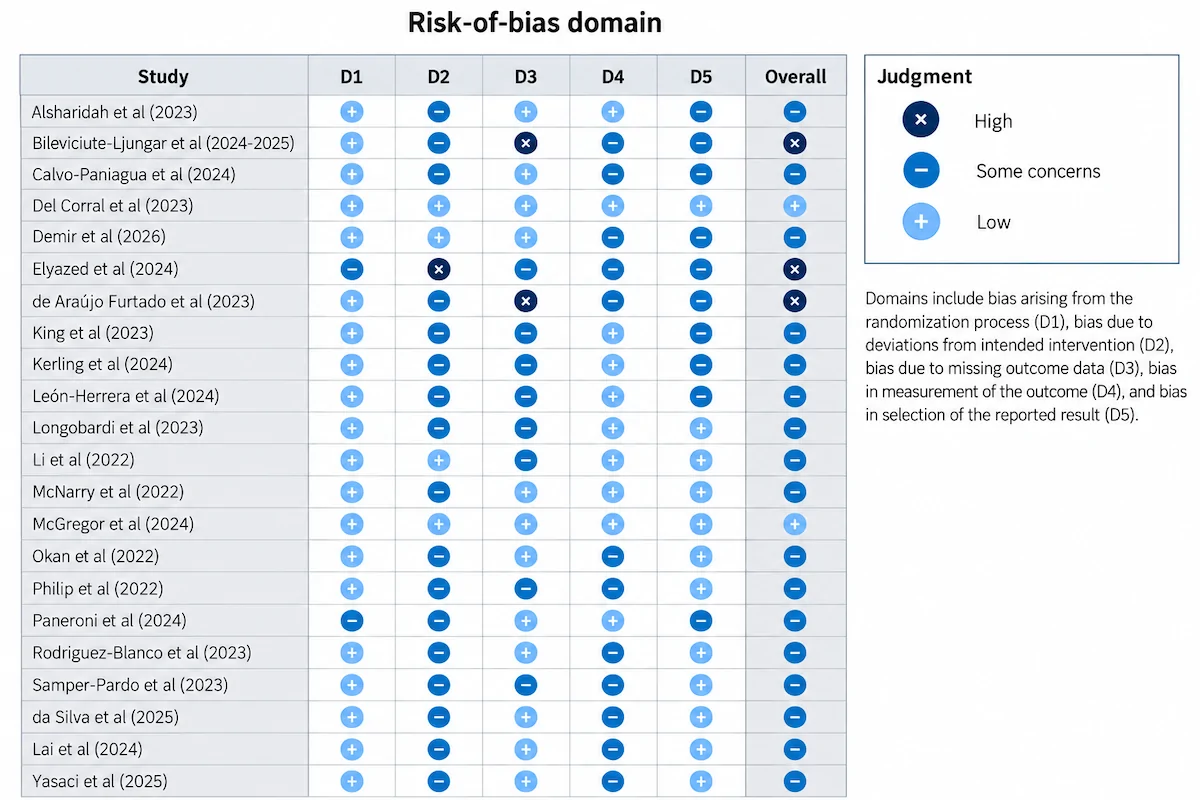
Risk of bias assessed using the Cochrane Risk of Bias tool (RoB 2).

### Health-Related Quality of Life

HRQoL was the primary outcome used to evaluate the overall efficacy of telerehabilitation. A total of 16 RCTs involving 1667 participants (819 in the intervention group and 848 in the control group) were included in the meta-analysis. In the pooled analysis using a random-effects model with HKSJ adjustment, telerehabilitation was associated with a statistically significant improvement in HRQoL compared with conventional care. The overall SMD was 1.26 (95% CI 0.07-2.45; *P*=.04). However, substantial heterogeneity was observed across the studies (*I*²=94.8%; τ²=4.63; *P*<.001; 95% PI –3.52 to 6.05), indicating considerable variability in the magnitude of the effect.

To investigate whether the delivery mode influenced efficacy, we performed a subgroup analysis stratified by delivery mode. The effect sizes for synchronous (k=7; SMD 1.53, 95% CI –0.98 to 4.05), asynchronous (k=6; SMD 1.03, 95% CI –1.38 to 3.44), and hybrid (k=3; SMD 1.14, 95% CI –2.53 to 4.81) telerehabilitation were comparable. The test for subgroup differences was not statistically significant (χ²_2_=0.14; *P*=.93), suggesting that the delivery mode was not a primary source of the observed heterogeneity. Notably, while the overall effect was significant, the confidence intervals for all individual subgroups crossed zero, likely attributable to the reduced sample size within subgroups and the conservative nature of the HKSJ adjustment (Figure 3) [42,45,46,48,50,52,54-59,61-63].

**Figure 3:**
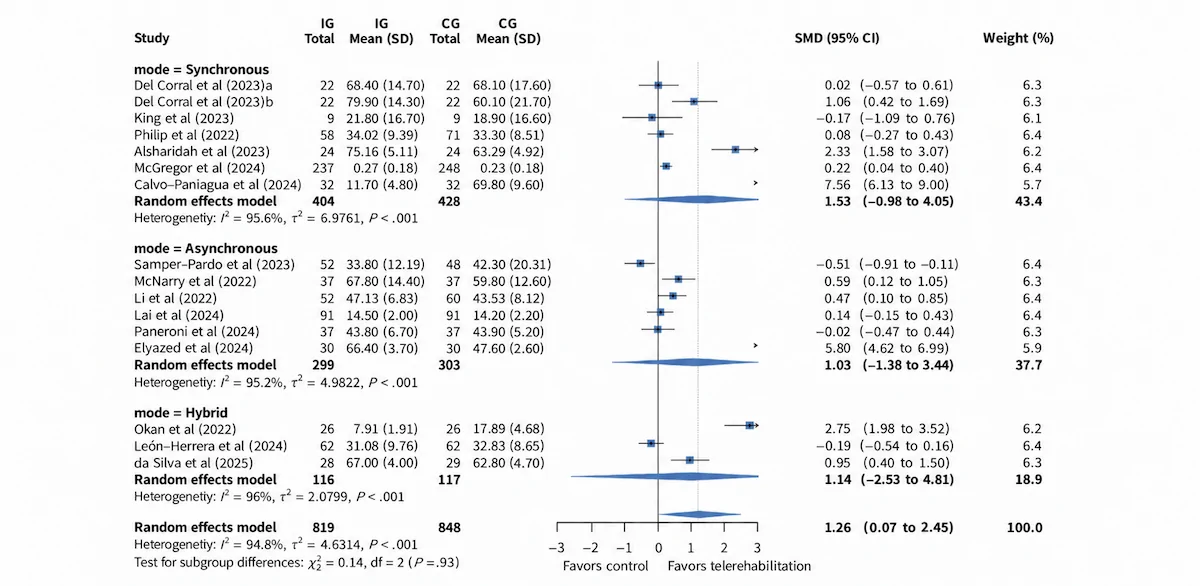
Forest plot of standardized mean differences for health-related quality of life comparing telerehabilitation with conventional care. Results are stratified by delivery. A random-effects model was applied. CG: control group; IG: intervention group; SMD: standardized mean difference.

We further explored potential sources of heterogeneity in HRQoL by participant age, time since diagnosis, and intervention duration (Table 4). None of these factors showed statistically significant subgroup differences (age, *P*=.11; time since diagnosis, *P*=.43; duration, *P*=.28), and substantial heterogeneity remained within all strata (*I*^2^=88%-97%). Although point estimates tended to be larger among older participants, those with a shorter time since diagnosis, and in shorter duration programs, confidence intervals were wide and largely overlapping. These findings suggest that the examined characteristics did not meaningfully explain the observed heterogeneity.

**Table 4:** Subgroup analyses of health-related quality of life by patient and intervention characteristics^a^.

Subgroup			Studies, n		SMD^b^ (95% CI)		*P* value		*I*^2^ (%)		*P* value for subgroup difference	
**Age (years)**												.11
	<50	8		0.40 (−0.33 to 1.13)		.23		88.3				
	≥50	8		2.15 (−0.29 to 4.59)		.08		97.0				
**Time since diagnosis (months)**												.43
	<6	6		1.87 (−0.45 to 4.19)		.09		96.6				
	≥6	9		0.91 (−0.97 to 2.79)		.30		94.0				
**Intervention duration (weeks)**												.28
	<8	6		2.14 (−0.84 to 5.12)		.12		96.9				
	≥8	10		0.75 (−0.52 to 2.01)		.22		92.8				

^a^Values are pooled SMDs with 95% CIs from random-effects meta-analysis with Hartung-Knapp adjustment. Subgroup differences were tested using Cochran Q.

^b^SMD: standardized mean difference.

### Functional Aerobic Capacity (6MWT)

Functional aerobic capacity was evaluated using the 6MWT across 10 RCTs involving 470 participants (Figure 4) [42,45,47-50,54,57,60]. In the overall pooled analysis using a random-effects model, telerehabilitation demonstrated a statistically significant improvement in functional aerobic capacity compared with conventional care (MD 77.79, 95% CI 30.44-125.14; *P*=.001). Although substantial heterogeneity was observed, a subgroup analysis stratified by delivery mode revealed comparable effects across synchronous (k=6; MD 77.70, 95% CI –25.31 to 180.71), asynchronous (k=3; MD 87.63, 95% CI 58.49-116.77), and hybrid (k=1; MD 59.07, 95% CI 25.82-92.32) interventions. The test for subgroup differences was not significant (χ^2^_2_=2.47; *P*=.29), suggesting that the efficacy of telerehabilitation in improving aerobic capacity did not differ significantly by delivery mode.

**Figure 4:**
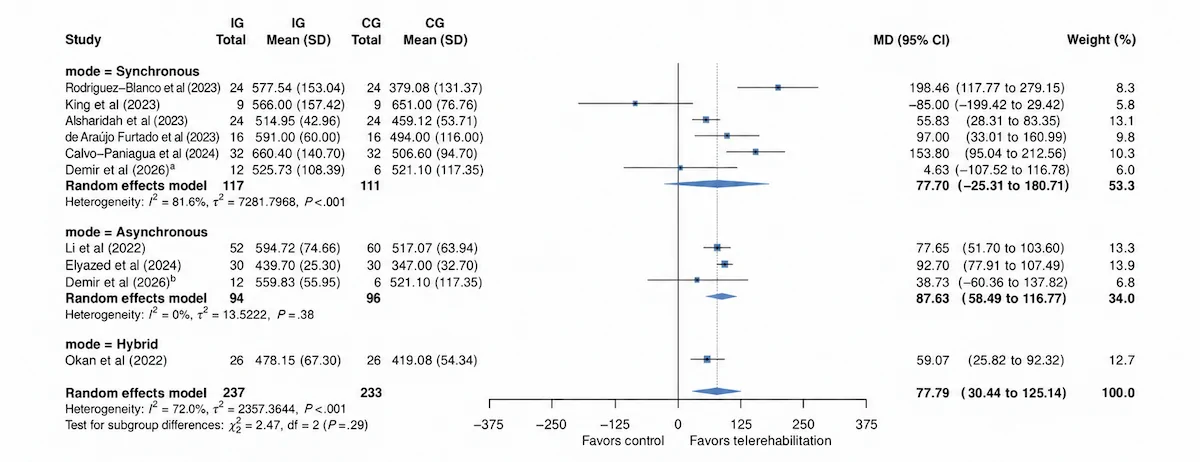
Forest plot of mean differences for functional aerobic capacity comparing telerehabilitation with conventional care. Results are stratified by delivery. A random-effects model was applied. CG: control group; IG: intervention group; MD: mean difference.

### Lower-Limb Muscle Strength (STS)

Lower-limb function was assessed with the STS in 10 RCTs (610 participants). The overall pooled effect did not reach significance (SMD 0.55, 95% CI –0.15 to 1.25; *P*=.12), with substantial heterogeneity (*I*^2^=88.5%). In the subgroup analysis by delivery mode, none of the 3 subgroups was individually significant (synchronous, k=5, SMD 0.53, 95% CI –0.22 to 1.27; asynchronous, k=2, SMD –0.13, 95% CI –0.62 to 0.35; hybrid, k=3, SMD 1.15, 95% CI –2.83 to 5.14). Although the test for subgroup differences was significant (χ^2^_2_=7.8; *P*=.02), the subgroup estimates were imprecise and within-subgroup heterogeneity was high, most notably in the hybrid group (*I*²=96.3%), where the pooled estimate was strongly influenced by a single trial. This subgroup difference should therefore be interpreted with caution (Figure 5) [46,50,52,53,59-62,64].

**Figure 5:**
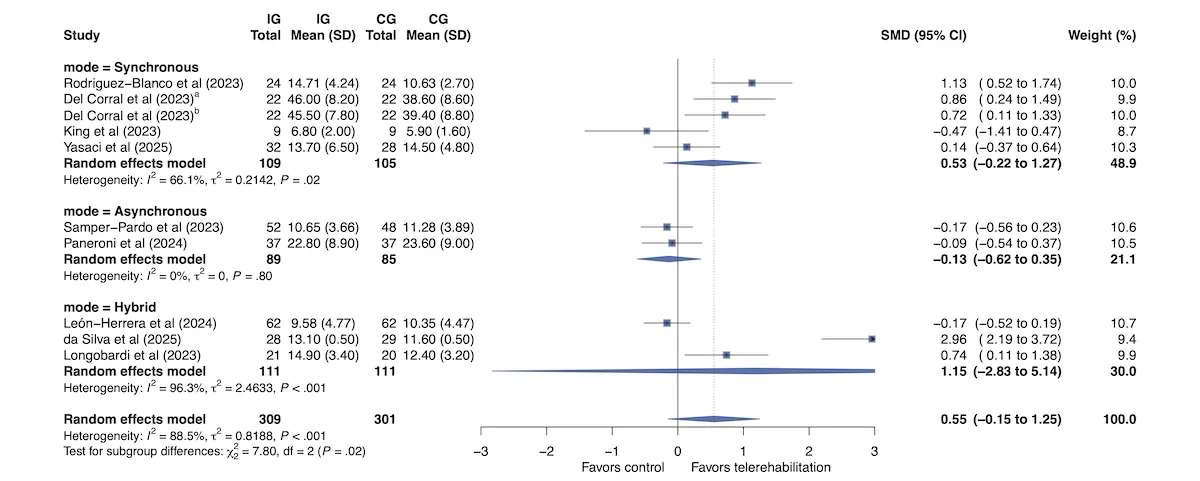
Forest plot of standardized mean differences for lower-limb muscle comparing telerehabilitation with conventional care. Results are stratified by delivery. A random-effects model was applied. CG: control group; IG: intervention group; SMD: standardized mean difference.

### Dyspnea

Dyspnea was evaluated in 9 RCTs involving 537 participants. In the pooled analysis, telerehabilitation yielded a statistically significant reduction in dyspnea compared with conventional care (SMD 1.95, 95% CI 0.60-3.31; *P*=.005). Subgroup analysis stratified by delivery mode showed that the synchronous intervention (k=5) achieved a large effect size (SMD 1.72, 95% CI –0.36 to 3.81), and the asynchronous intervention (k=3) similarly demonstrated positive outcomes (SMD 1.91, 95% CI –4.05 to 7.88). Additionally, the hybrid intervention (k=1) showed a substantial effect (SMD 3.26, 95% CI 2.42-4.11). The test for subgroup differences was not statistically significant (χ²_2_=3.6; *P*=.16), indicating that the delivery mode did not significantly alter the therapeutic benefit (Figure 6) [45,47,55,57-60,64].

**Figure 6:**
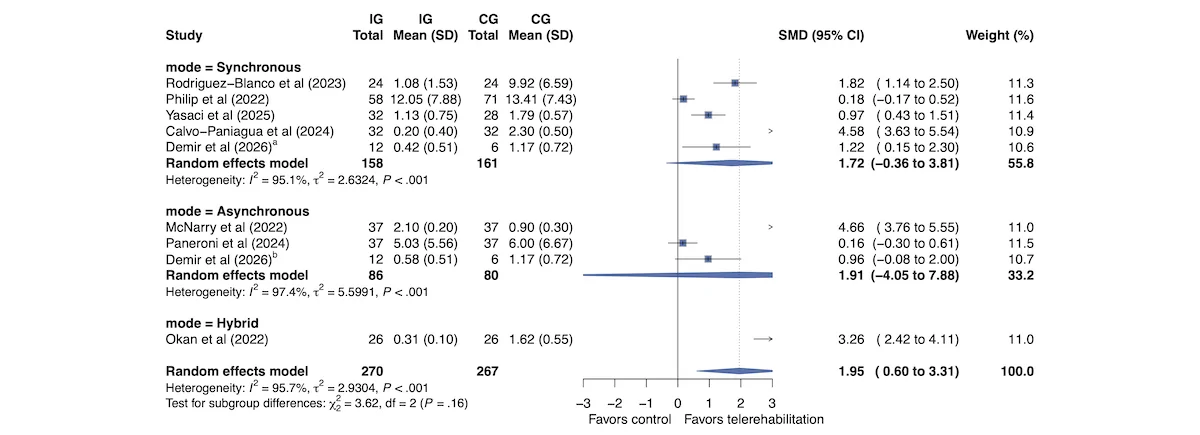
Forest plot of standardized mean differences for dyspnea comparing telerehabilitation with conventional care. Results are stratified by delivery mode. A random-effects model was applied. CG: control group; IG: intervention group; SMD: standardized mean difference.

### Fatigue

Fatigue was evaluated in 6 RCTs (n=236). The pooled analysis indicated that telerehabilitation yielded a statistically significant reduction in fatigue compared with conventional care (SMD 0.89, 95% CI 0.16-1.62; *P*=.02) (Figure 7) [47,48,50,59,60].

**Figure 7:**
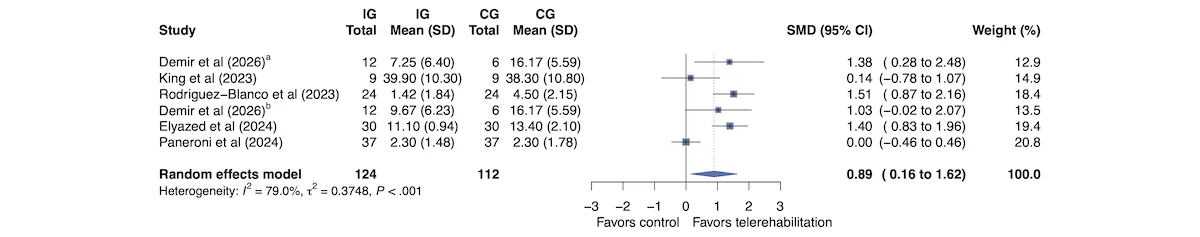
Forest plot of standardized mean differences for fatigue comparing telerehabilitation with conventional care. A random-effects model was applied. CG: control group; IG: intervention group; SMD: standardized mean difference.

### Anxiety and Depression Symptoms

Five RCTs reported anxiety and depression outcomes. Because these were assessed with different instruments (the HADS total score in 2 trials, the HADS anxiety subscale in 2, and the GAD-7 in 1), effects were pooled as SMDs. The random-effects meta-analysis showed no significant difference between telerehabilitation and control interventions (SMD 0.12, 95% CI –0.13 to 0.38; P=.36). Between-study heterogeneity was low to moderate (I²=43.8%, P=.13). Owing to the limited number of studies, no subgroup analyses were performed (Figure 8) [52,56,58,61,64].

**Figure 8:**
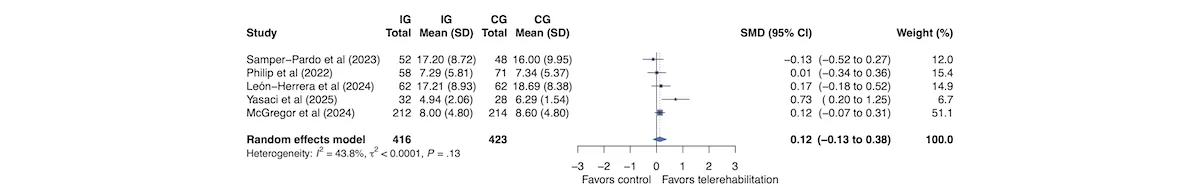
Forest plot of standardized mean differences for anxiety and depression symptoms comparing telerehabilitation with conventional care. A random-effects model was applied. CG: control group; IG: intervention group; SMD: standardized mean difference.

### Overview of Effects Across Outcomes

An overview forest plot was used to summarize pooled effects across outcome domains. Overall, telerehabilitation showed a consistent favorable direction of effect for most physical and functional outcomes, with the largest effects observed for dyspnea and HRQoL, followed by improvements in aerobic capacity (6MWT) and fatigue.

In contrast, mental health outcomes assessed by symptoms of anxiety and depression showed small and nonsignificant effects, with estimates centered around the null. Heterogeneity varied across outcomes and was generally more pronounced for physical and respiratory measures. Collectively, these findings suggest that telerehabilitation confers more robust benefits in physical and functional domains than in psychological outcomes (Figure 9).

**Figure 9:**
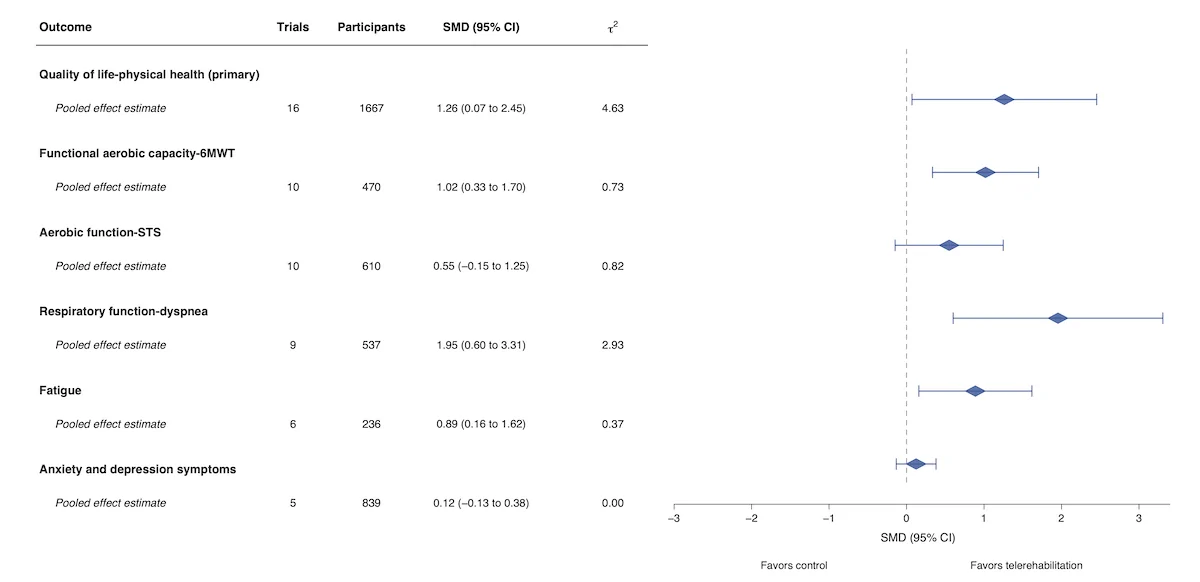
Summary forest plot of pooled standardized mean differences for primary and secondary outcomes comparing telerehabilitation with conventional care. A random-effects model was applied. 6MWT: 6-minute walk test; SMD: standardized mean difference; STS: sit-to-stand test.

### Sensitivity Analysis

Given the high heterogeneity across outcomes, a leave-one-out sensitivity analysis was performed to test the stability of the pooled results. Detailed results are shown in Multimedia Appendix 4. The analysis confirmed the robustness of the primary findings, as omitting any single trial did not alter the statistical significance for HRQoL, 6MWT, or STS. For Fatigue and the anxiety and depression outcome, specific outliers were identified. Excluding 2 studies [59,64] significantly reduced heterogeneity (*I*^2^ dropped to 39.0% for fatigue and 0% for anxiety and depression) while maintaining the direction of the therapeutic effect, thereby validating the reliability of our conclusions.

### Publication Bias Test

Publication bias was assessed exclusively for the primary outcome (HRQoL) because other outcomes did not meet the recommended threshold of included studies (k<10) for reliable testing. The funnel plot for HRQoL (Figure 10) displays a symmetrical distribution of studies, and Egger linear regression test confirmed no evidence of significant small-study effects (*t*=–0.78; *P*=.46), indicating a low risk of publication bias.

**Figure 10:**
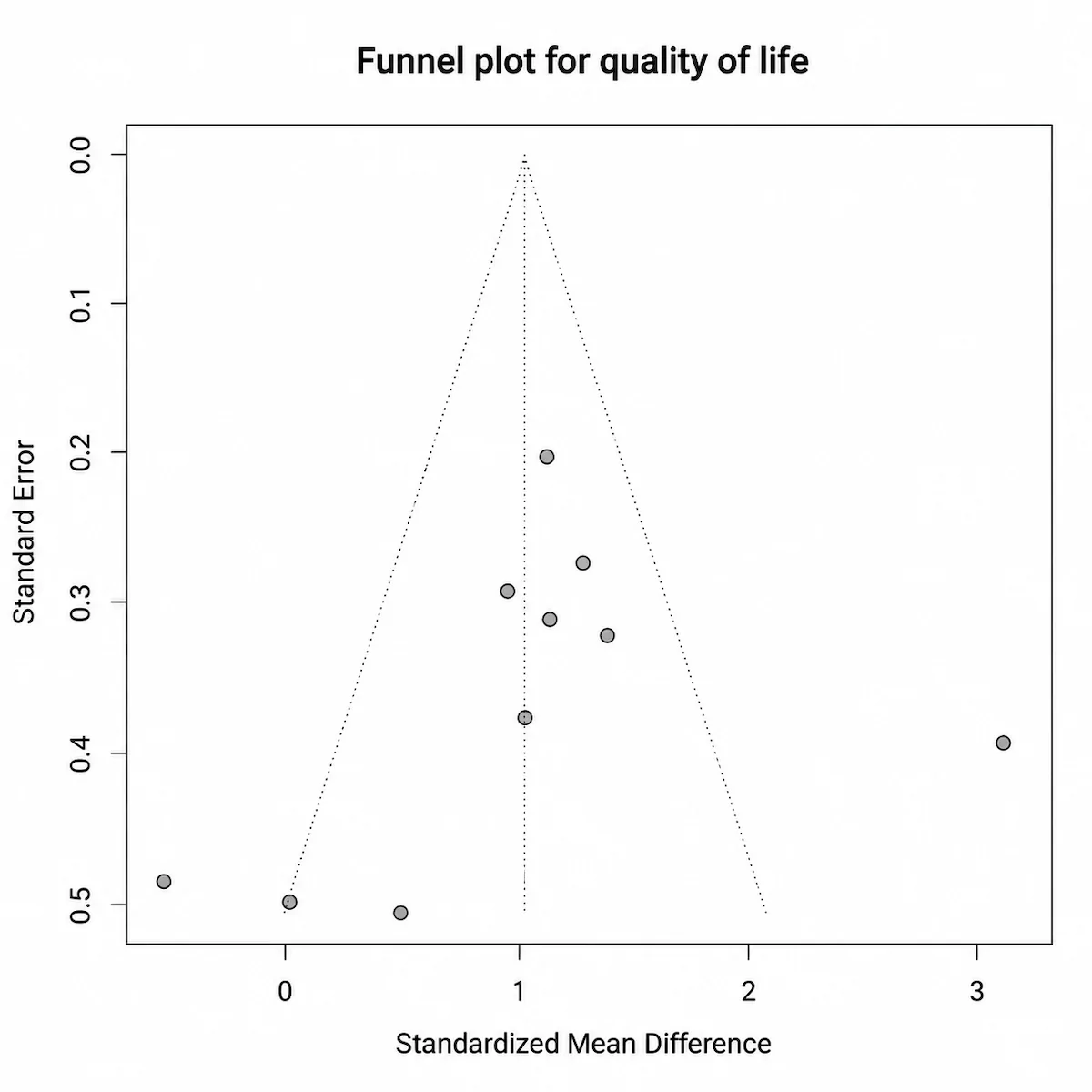
Funnel plot for small-study effects in health-related quality of life meta-analysis.

### Certainty of Evidence

According to the GRADE approach, the certainty of evidence was rated as low for all outcomes (Multimedia Appendix 5). For HRQoL, functional capacity (6MWT and STS), dyspnea, and fatigue, the certainty was downgraded primarily due to risk of bias and inconsistency, reflecting methodological limitations and substantial between-study heterogeneity. In contrast, the certainty of evidence for symptoms of anxiety and depression was downgraded due to risk of bias and imprecision, owing to the limited number of contributing trials and relatively wide confidence intervals. No serious concerns regarding indirectness or publication bias were identified across outcomes. Overall, these findings indicate that the pooled estimates should be interpreted cautiously.

## Discussion

### Principal Results

This systematic review and meta-analysis of 22 RCTs evaluated the effects of telerehabilitation in adults with PCC. Our synthesis indicates that telerehabilitation was associated with significant improvements in HRQoL, functional aerobic capacity (6MWT), dyspnea, and fatigue compared with control group. From a clinical perspective, the observed improvement in functional aerobic capacity also appears meaningful: the pooled increase in 6-minute walk distance (MD 77.79 meters) exceeded the minimal clinically important difference of approximately 30 meters reported for chronic respiratory disease [33], suggesting that the effect is likely to be clinically relevant. This estimate is broadly consistent with a recent updated meta-analysis, which reported a mean improvement of 85.83 meters in 6-minute walk distance [28]. In contrast, HRQoL, dyspnea, and fatigue were synthesized using SMDs across multiple instruments and therefore could not be interpreted against a single minimal clinically important difference threshold. Beyond these overall benefits, the present findings suggest that the role of supervision in telerehabilitation may vary according to the targeted outcome. Stratified analyses suggested that the benefits for general aerobic capacity and HRQoL were broadly comparable across delivery modes, whereas a subgroup difference favoring synchronous delivery was observed for lower-limb muscle strength. These subgroup findings were exploratory and should be interpreted with caution.

Because the subgroup differences for HRQoL and 6MWT were not significant, our data do not suggest that continuous real-time supervision is required to improve general aerobic capacity, although the limited power of these comparisons means equivalence cannot be assumed. Asynchronous protocols may be sufficient for these outcomes, possibly because they allow patients to incorporate low-to-moderate intensity activity into daily routines, consistent with evidence that structured home-based rehabilitation can improve endurance regardless of direct supervision [76,77]. For lower-limb strength (STS), by contrast, a significant subgroup difference favored synchronous delivery; however, neither modality reached significance on its own, so synchronous supervision may play a more important role in strength training rather than being essential. A possible explanation is that real-time monitoring helps therapists gauge exertion and progress intensity safely [78], while supervised sessions may support adherence and correct exercise technique [79]. These factors may be particularly important for strength training, which requires adequate progression and training intensity to achieve meaningful gains.

Data on adherence and patient satisfaction were reported in limited studies, preventing a quantitative meta-analysis. Nevertheless, these factors are clinically relevant, as satisfaction is a key predictor of long-term adherence and outcomes [80]. Based on current evidence, the mode of delivery appears to influence patient engagement [76]. Asynchronous telerehabilitation provides temporal flexibility, allowing patients with fatigue to adjust exercise timing to their energy levels. This feasibility likely contributed to the QoL improvements observed in the self-managed group. Conversely, synchronous modes involve real-time interaction that may strengthen the therapeutic alliance and accountability. This supervision may help sustain participant engagement and adequate training intensity. A hybrid approach combining asynchronous delivery with periodic synchronous supervision may therefore provide a practical balance between accessibility and clinical support, although this hypothesis requires confirmation in future studies.

With respect to persistent symptoms, telerehabilitation was associated with significant improvements in both dyspnea and fatigue. The heterogeneity in dyspnea outcomes is consistent with prior evidence syntheses and may reflect differences in intervention focus [18]. Programs that included dyspnea-targeted components, such as inspiratory muscle training, appeared more likely to yield clinically meaningful benefit. The reduction in fatigue represented a large effect size (SMD 0.89), underscoring its clinical importance and aligning with accumulating evidence supporting structured exercise for PCC [26]. This benefit may reflect the counteracting of physical deconditioning through pacing and gradual, individualized reconditioning, without worsening symptoms. Conversely, consistent with prior evidence, we found no significant impact of physical telerehabilitation on symptoms of anxiety and depression [81]. Anxiety in PCC may involve complex neuroinflammatory and psychosocial mechanisms [82], which physical rehabilitation alone may not sufficiently address. Among the studies that reported adverse events, none were serious or intervention-related, although adverse event reporting was inconsistent across trials. The available evidence therefore suggests that telerehabilitation appears to be safe under the conditions evaluated, but trials with standardized safety reporting are warranted. This underscores the need for integrated, multicomponent interventions that combine physical training with structured psychological strategies, such as cognitive behavioral approaches.

Substantial heterogeneity was observed across several pooled outcomes, particularly HRQoL, and the findings should therefore be interpreted with caution. Subgroup analyses by delivery mode, participant age, time since diagnosis, and intervention duration did not identify statistically significant subgroup differences, and substantial residual heterogeneity remained within strata. Several other factors may contribute. First, HRQoL was assessed using different instruments, including generic measures (eg, SF-36 and EQ-5D) and disease-specific tools such as the St George Respiratory Questionnaire, which differ in construct, scaling, and responsiveness; such variation in measurement may contribute to between-study variability. Second, the populations ranged from mild symptoms to survivors of critical illness, and from community to posthospital settings, implying different baseline impairment and recovery trajectories. Third, the interventions differed in content, progression, and exercise dose, from single-component breathing or inspiratory muscle training to multicomponent aerobic and resistance programs (Table 2); their predominantly multicomponent nature limited the feasibility of a clean subgroup analysis by individual component, so these differences were considered narratively. Because none of these factors were formally tested as moderators, they should be regarded as plausible explanations rather than definitive sources of heterogeneity. Practically, this uncertainty is reflected in the wide PI, indicating that the true effect may vary substantially across future settings and patient populations, whose broader dynamics have also been characterized through population-level pandemic modeling [83]. These findings should therefore be interpreted as average effects across heterogeneous contexts rather than precise estimates applicable to every patient.

Although subgroup analysis by intervention component was not feasible because of the limited number of studies, the interventions can be broadly summarized using the frequency, intensity, time, and type framework (Table 2). Most programs prescribed exercise 2-6 times per week for 20-90 minutes over 2-16 weeks. Exercise intensity was generally individualized using Borg ratings of perceived exertion, percentages of maximal heart rate or heart rate reserve, American College of Sports Medicine recommendations, or, for inspiratory muscle training, percentages of maximal inspiratory pressure. Interventions ranged from single-component breathing or inspiratory muscle training to multicomponent programs combining aerobic and resistance exercise. Overall, these findings suggest that individualized exercise programs delivered regularly over several weeks may be beneficial, although considerable variation remained in exercise intensity and program composition, and an optimal exercise prescription cannot be established from the current evidence.

### Limitations

This systematic review and meta-analysis has several limitations that warrant consideration. First, substantial heterogeneity was present across the main pooled outcomes and, as discussed previously, likely reflected differences in measurement instruments, clinical populations, and intervention characteristics; although we used random-effects models and subgroup analyses, much of it remained unexplained. Second, the evidence base is constrained by the nature of rehabilitation interventions, where blinding of participants and therapists was largely infeasible, introducing potential performance and detection biases, particularly for subjective patient-reported outcomes such as HRQoL and dyspnea. Moreover, several key outcomes, including HRQoL, fatigue, and anxiety and depression, were assessed using patient-reported measures and are therefore inherently subjective. These outcomes may be influenced by participants’ expectations, mood, social context, and the absence of blinding. Accordingly, they should be interpreted together with the objective functional outcomes and in light of the overall certainty of evidence. Third, the limited number of studies in certain subgroups constrained statistical power. Nevertheless, these exploratory analyses were included to discern potential trends regarding delivery modes; consequently, the findings should be interpreted as hypothesis-generating rather than definitive. Finally, most included trials had relatively short follow-up periods (typically 4-12 weeks) and often lacked rigorous assessments of patients’ digital literacy, limiting our ability to determine the long-term sustainability of benefits and the generalizability of findings to older or less tech-savvy populations in real-world settings.

### Future Perspectives

Collectively, these findings are consistent with a tentative stepped-care approach, although they should be regarded as hypothesis-generating given the low certainty of evidence and the wide, overlapping confidence intervals of the subgroup estimates. For most patients aiming to recover general fitness and quality of life, asynchronous telerehabilitation appears to be a reasonable and scalable option, given its broadly comparable effects and lower resource demands. For patients with marked muscle weakness or those requiring closer safety monitoring, synchronous supervision may be preferable. These suggestions require confirmation in adequately powered trials. Considering the limitations of the current evidence base, future research should prioritize 3 directions. First, to address the null effects observed for mental health outcomes, telerehabilitation protocols should move beyond a solely physical rehabilitation paradigm. The integration of structured physical exercise with remotely delivered psychological components, such as online cognitive behavioral therapy or mindfulness-based interventions, may offer a more comprehensive approach to the neuropsychiatric burden of PCC, consistent with emerging multidisciplinary models proposed in recent literature [81]. Second, given that most included trials were characterized by relatively short follow-up periods, there is a clear need to examine the long-term sustainability of observed benefits, particularly for outcomes such as muscle strength and functional capacity. Future studies should assess whether intervention effects are maintained at 6- and 12-month follow-up and explore whether incorporating periodic synchronous booster sessions into predominantly asynchronous programs can enhance adherence and prevent functional regression over time. Finally, although asynchronous telerehabilitation is often assumed to be more cost-efficient than conventional face-to-face rehabilitation [84], robust economic evaluations remain scarce. Rigorous cost-effectiveness and cost-utility analyses are therefore required to quantify potential economic advantages and to inform health policy decisions, particularly with respect to the inclusion of telerehabilitation within insurance reimbursement frameworks.

### Conclusions

This review synthesized randomized evidence on telerehabilitation for adults with PCC, with a focus on HRQoL and delivery mode. Telerehabilitation may improve HRQoL, aerobic capacity, dyspnea, and fatigue, whereas no clear benefit was observed for symptoms of anxiety or depression. Evidence for lower-limb strength remained inconclusive, and any added value of synchronous supervision should be interpreted cautiously. Given the limited certainty of evidence for several outcomes and substantial heterogeneity across studies, telerehabilitation should be viewed as a promising and accessible rehabilitation option rather than an established standard of care. Future trials should use standardized outcomes, longer follow-up, and prespecified analyses of delivery mode to confirm benefits and clarify which patients are most likely to benefit.

## Data Availability

All data generated or analyzed during this study are included in this published article and its multimedia appendices. The data analyzed in this systematic review and meta-analysis were extracted from previously published studies, which are cited in the manuscript. No new primary data were generated.

## Appendix

Multimedia Appendix 1 PRISMA 2020 checklist.

Multimedia Appendix 2 Full electronic search strategy for each database.

Multimedia Appendix 3 Outcome measures, instruments, scoring direction, and orientation in the meta-analysis.

Multimedia Appendix 4 Leave-One-Out Meta-Analysis for all outcomes.

Multimedia Appendix 5 The GRADE assessments.

## Abbreviations

GRADE: Grading of Recommendations Assessment, Development and Evaluation
HADS: Hospital Anxiety and Depression Scale
HKSJ: Hartung-Knapp-Sidik-Jonkman
HRQoL: health-related quality of life
MD: mean difference
MoCA: Montreal Cognitive Assessment
PCC: post–COVID-19 condition
PI: prediction interval
PICOS: Population, Intervention, Comparison, Outcome, and Study design
PRISMA: Preferred Reporting Items for Systematic Reviews and Meta-Analyses
RCT: randomized controlled trial
RoB 2: Cochrane Risk of Bias 2 tool
6MWT: 6-minute walk test
SF-36: 36-Item Short Form Health Survey
SMD: standardized mean difference
STS: sit-to-stand test
