# Supplementary material for: Effects of Telerehabilitation on Quality of Life, Physical Capacity, and Symptom Burden Within the Post–COVID-19 Condition: Systematic Review and Meta-Analysis of Randomized Controlled Trials
Source: JMIR Rehabil Assist Technol. 2026 Oct 27;13:e95368. doi: 10.2196/95368 (PMC13752595; doi:10.2196/95368)
Supplement: Supplementary file 2 [file rehab_v13i1e95368_app5.pdf]

Author(s):

Question: Telerehabilitation compared to Usual care for Post–COVID-19 Condition

Setting:

Bibliography:

Certainty assessment							№ of patients		Effect		Certainty	Importance
№ of studies	Study design	Risk of bias	Inconsistency	Indirectness	Imprecision	Other considerations	Telerehabilitation	Usual care	Relative (95% CI)	Absolute (95% CI)		
**HRQoL**												
16	randomised trials	serious[a]	serious[b]	not serious	not serious	none[g]	819	848	-	SMD **1.26 SD higher** (0.07 higher to 2.45 higher)	⨁⨁◯◯ Low[a,b]	CRITICAL
**6MWT**												
10	randomised trials	serious[c]	serious	not serious[d]	not serious	none[g]	237	233	-	MD **77.79 m higher** (30.44 higher to 125.14 higher)	⨁⨁◯◯ Low[c,d]	CRITICAL
**STS**												
10	randomised trials	serious[e]	serious[e]	not serious[e]	not serious	none[g]	309	301	-	SMD **0.55 SD higher** (0.15 lower to 1.25 higher)	⨁⨁◯◯ Low[e]	CRITICAL
**Dyspnea**												
9	randomised trials	serious[a]	serious[c]	not serious	not serious	none[g]	270	267	-	SMD **1.95 SD higher** (0.6 higher to 3.31 higher)	⨁⨁◯◯ Low[a,c]	CRITICAL
**Fatigue**												

Certainty assessment							№ of patients		Effect		Certainty	Importance
№ of studies	Study design	Risk of bias	Inconsistency	Indirectness	Imprecision	Other considerations	Telerehabilitation	Usual care	Relative (95% CI)	Absolute (95% CI)		
6	randomised trials	serious[a]	serious[b]	not serious	not serious	none[g]	124	112	-	SMD **0.89 SD higher** (0.16 higher to 1.62 higher)	⨁⨁◯◯ Low[a,b]	CRITICAL
**Anxiety and depression symptoms**												
5	randomised trials	serious[a]	not serious	not serious	serious[f]	none[g]	416	423	-	SMD **0.12 SD higher** (0.13 lower to 0.38 higher)	⨁⨁◯◯ Low[a,f]	IMPORTANT

CI: confidence interval; **MD:** mean difference; **SMD:** standardised mean difference

Explanations

a. Most included trials involved exercise-based or rehabilitation interventions, for which blinding of participants and personnel was not feasible. In addition, quality of life outcomes were primarily assessed using self-reported instruments, introducing potential performance and detection bias. Although most studies were rated as having "some concerns" rather than high risk of bias, these limitations warranted downgrading the certainty of evidence.

b. Substantial statistical heterogeneity was observed across studies ($I^2 \approx 95\%$), indicating considerable variability in effect estimates. Although subgroup analyses did not reveal statistically significant differences between intervention modes and the direction of effects was largely consistent, the magnitude of effects varied substantially across trials, justifying downgrading for inconsistency.

c. Most included trials involved exercise-based rehabilitation interventions, in which blinding of participants and personnel was not feasible. Although the 6-minute walk test is an objective performance-based outcome, lack of blinding and potential deviations from intended interventions introduce concerns regarding performance bias. Most studies were judged to have some concerns rather than high risk of bias.

d. Substantial statistical heterogeneity was observed in the pooled analysis ($I^2 \approx 72\%$), indicating variability in effect magnitude across studies. Although subgroup analyses did not demonstrate statistically significant differences between intervention delivery modes and the direction of effects consistently favored telerehabilitation, the magnitude of improvement varied across trials, warranting downgrading for inconsistency.

e. Most included studies evaluated exercise-based rehabilitation interventions, for which blinding of participants and personnel was not feasible. Although the test represents a performance-based and relatively objective outcome, lack of blinding and potential deviations from intended interventions introduce concerns regarding performance bias. Overall, most studies were judged to have some concerns rather than high risk of bias.

f. The pooled effect estimate was small and not statistically significant, with confidence intervals crossing the line of no effect. In addition, the total sample size was limited, and the observed effects did not reach a magnitude considered clinically meaningful, warranting downgrading for imprecision.

g. Publication bias was formally assessed only for HRQoL, the single outcome with ≥10 studies (funnel plot and Egger test showed no evidence of small-study effects). For the remaining outcomes it could not be reliably assessed owing to the small number of trials; "undetected" therefore reflects the absence of detected bias rather than a formal confirmation of its absence.
