# Supplementary material for: Effects of Telerehabilitation on Quality of Life, Physical Capacity, and Symptom Burden Within the Post–COVID-19 Condition: Systematic Review and Meta-Analysis of Randomized Controlled Trials
Source: JMIR Rehabil Assist Technol. 2026 Oct 27;13:e95368. doi: 10.2196/95368 (PMC13752595; doi:10.2196/95368)
Supplement: Supplementary file 3 [file rehab_v13i1e95368_app2.docx]

**Multimedia Appendix 2**

**Pubmed/MEDLINE:**

(("Telerehabilitation"[Mesh] OR telerehabilitation OR "Tele-rehabilitation" OR "Tele rehabilitation" OR "Remote Rehabilitation" OR "Rehabilitation, Remote" OR "Virtual Rehabilitation*" OR "Rehabilitation, Virtual" OR "Digital Rehabilitation" OR "Home-based Rehabilitation" OR "Home based rehabilitation" OR telehealth OR "Telemedicine"[Mesh] OR telemedicine OR telemonitoring OR teletherapy OR teletreatment* OR telepractice OR "Video Conferencing"[Mesh] OR videoconferenc* OR "Virtual Conferenc*" OR eHealth OR mHealth OR "Mobile Health" OR "Internet-Based Intervention*" OR "Web-Based Intervention*" OR exergam* OR "Exergaming"[Mesh] OR "Virtual Reality Exercise")

AND

("Post-Acute COVID-19 Syndrome"[Mesh] OR "Long COVID" OR "Long-COVID" OR "Post COVID Condition" OR "Post-COVID Condition" OR "Post–COVID-19 Condition" OR "Post-COVID-19 condition" OR "Post Acute COVID" OR "Post-Acute COVID" OR "Post-acute COVID-19" OR "Post-acute SARS-CoV-2" OR "Post COVID Syndrome" OR "Post-COVID Syndrome" OR "Post-COVID-19 Syndrome" OR "Chronic COVID" OR "Persistent COVID" OR "Persistent Symptoms after COVID-19" OR "COVID-19 Sequelae" OR "COVID-19 Complications" OR "SARS-CoV-2 Sequelae" OR "COVID-19"[Mesh])

AND

("Randomized Controlled Trial"[Publication Type] OR "Controlled Clinical Trial"[Publication Type] OR randomized OR randomised OR randomly OR trial OR RCT)

AND

(adult[MeSH Terms] OR adults OR "18 years" OR "≥18 years"))

**Web of science:**

TS=(Telerehabilitation OR telerehabilitation OR Telerehabilitations OR "Tele-rehabilitation" OR "Tele rehabilitation" OR "Remote Rehabilitation" OR "Rehabilitation, Remote" OR "Virtual Rehabilitation*" OR "Rehabilitation, Virtual" OR "Digital Rehabilitation" OR "Home-based Rehabilitation" OR "Home based rehabilitation" OR telehealth OR Telemedicine OR telemedicine OR telemonitoring OR teletherapy OR teletreatment* OR Telepractice OR videoconferenc* OR "Virtual Conferenc*" OR eHealth OR mHealth OR "Mobile Health" OR "Internet-Based Intervention*" OR "Web-Based Intervention*" OR Exergaming OR exergaming OR exergam* OR "Virtual Reality Exercise")

AND

TS=("Long COVID" OR "Long-COVID" OR "Post COVID Condition" OR "Post-COVID Condition" OR "Post–COVID-19 Condition" OR "Post-COVID-19 condition" OR "Post Acute COVID" OR "Post-Acute COVID" OR "Post-acute COVID-19" OR "Post-acute SARS-CoV-2" OR "Post COVID Syndrome" OR "Post-COVID Syndrome" OR "Post-COVID-19 Syndrome" OR "Post-Acute COVID-19 Syndrome" OR "Chronic COVID" OR "Persistent COVID" OR "Persistent Symptoms after COVID-19" OR "COVID-19 Sequelae" OR "COVID-19 Complications" OR "SARS-CoV-2 Sequelae" OR "Post COVID-19 sequelae")

AND

TS=(randomized OR randomised OR randomly OR "randomized controlled trial" OR "controlled clinical trial" OR RCT OR trial)

**Cochrane:**

((MeSH descriptor: [Telerehabilitation] explode all trees OR MeSH descriptor: [Telemedicine] explode all trees OR telerehabilitation OR "tele-rehabilitation" OR "tele rehabilitation" OR "remote rehabilitation" OR "virtual rehabilitation*" OR "digital rehabilitation" OR "home-based rehabilitation" OR telehealth OR telemedicine OR telemonitoring OR teletherapy OR teletreatment* OR telepractice OR videoconferenc* OR "virtual conferenc*" OR eHealth OR mHealth OR "mobile health" OR "internet-based intervention*" OR "web-based intervention*" OR exergam* OR "virtual reality exercise"):ti,ab,kw)

AND

((MeSH descriptor: [Post-Acute COVID-19 Syndrome] explode all trees OR "long COVID" OR "long-COVID" OR "post COVID condition" OR "post-COVID condition" OR "post-acute COVID" OR "post acute COVID" OR "post-COVID syndrome" OR "post-COVID-19 syndrome" OR "post-acute COVID-19" OR "chronic COVID" OR "persistent COVID" OR "COVID-19 sequelae" OR "SARS-CoV-2 sequelae" OR "persistent symptoms after COVID-19"):ti,ab,kw)

**Scopus：**

TITLE-ABS-KEY (telerehabilitation OR "tele-rehabilitation" OR "tele rehabilitation" OR "remote rehabilitation" OR "virtual rehabilitation*" OR "digital rehabilitation" OR "home-based rehabilitation" OR telehealth OR telemedicine OR telemonitoring OR teletherapy OR teletreatment* OR telepractice OR videoconferenc* OR "virtual conferenc*" OR ehealth OR mhealth OR "mobile health" OR "internet-based intervention*" OR "web-based intervention*" OR exergam* OR "virtual reality exercise")

AND

TITLE-ABS-KEY ("long covid" OR "post covid condition" OR "post-covid condition" OR "post-acute covid" OR "post covid syndrome" OR "post-covid-19 syndrome" OR "chronic covid" OR "persistent covid" OR "covid-19 sequelae" OR "sars-cov-2 sequelae" OR "persistent symptoms after covid-19")

AND

TITLE-ABS-KEY (randomized OR randomised OR randomly OR "randomized controlled trial" OR "controlled clinical trial" OR rct OR trial)

**Embase：**

('telerehabilitation'/exp OR 'telemedicine'/exp OR telerehabilitation OR 'tele-rehabilitation' OR 'tele rehabilitation' OR 'remote rehabilitation' OR 'virtual rehabilitation*' OR 'digital rehabilitation' OR 'home-based rehabilitation' OR telehealth OR telemedicine OR telemonitoring OR teletherapy OR teletreatment* OR telepractice OR videoconferenc* OR 'virtual conferenc*' OR ehealth OR mhealth OR 'mobile health' OR 'internet-based intervention*' OR 'web-based intervention*' OR exergam* OR 'virtual reality exercise'):ti,ab,kw

AND

('post acute covid 19 syndrome'/exp OR 'long covid' OR 'post covid condition' OR 'post-covid condition' OR 'post-acute covid' OR 'post covid syndrome' OR 'post-covid-19 syndrome' OR 'chronic covid' OR 'persistent covid' OR 'covid-19 sequelae' OR 'sars-cov-2 sequelae' OR 'persistent symptoms after covid-19'):ti,ab,kw

AND

('randomized controlled trial'/exp OR 'controlled clinical trial'/exp OR randomized OR randomised OR randomly OR trial OR rct):ti,ab,kw

**EBSCO:**

((MH "Telerehabilitation+") OR (MH "Telemedicine+") OR TI (telerehabilitation OR "tele-rehabilitation" OR "tele rehabilitation" OR "remote rehabilitation" OR "virtual rehabilitation*" OR "digital rehabilitation" OR "home-based rehabilitation" OR telehealth OR telemedicine OR telemonitoring OR teletherapy OR teletreatment* OR telepractice OR videoconferenc* OR "virtual conferenc*" OR ehealth OR mhealth OR "mobile health" OR "internet-based intervention*" OR "web-based intervention*" OR exergam* OR "virtual reality exercise") OR AB (telerehabilitation OR "tele-rehabilitation" OR "tele rehabilitation" OR "remote rehabilitation" OR "virtual rehabilitation*" OR "digital rehabilitation" OR "home-based rehabilitation" OR telehealth OR telemedicine OR telemonitoring OR teletherapy OR teletreatment* OR telepractice OR videoconferenc* OR "virtual conferenc*" OR ehealth OR mhealth OR "mobile health" OR "internet-based intervention*" OR "web-based intervention*" OR exergam* OR "virtual reality exercise"))

AND

((MH "Post-Acute COVID-19 Syndrome+") OR TI ("long covid" OR "post covid condition" OR "post-covid condition" OR "post-acute covid" OR "post covid syndrome" OR "post-covid-19 syndrome" OR "chronic covid" OR "persistent covid" OR "covid-19 sequelae" OR "sars-cov-2 sequelae" OR "persistent symptoms after covid-19") OR AB ("long covid" OR "post covid condition" OR "post-covid condition" OR "post-acute covid" OR "post covid syndrome" OR "post-covid-19 syndrome" OR "chronic covid" OR "persistent covid" OR "covid-19 sequelae" OR "sars-cov-2 sequelae" OR "persistent symptoms after covid-19"))

AND

(TI (randomized OR randomised OR randomly OR "randomized controlled trial" OR "controlled clinical trial" OR rct OR trial) OR AB (randomized OR randomised OR randomly OR "randomized controlled trial" OR "controlled clinical trial" OR rct OR trial))

**PEDro:**

telerehabilitation OR tele-rehabilitation OR "remote rehabilitation" OR telehealth OR telemedicine OR teletherapy OR telemonitoring

AND

"long covid" OR "post covid condition" OR "post-acute covid" OR "post covid syndrome" OR "post-covid-19 syndrome" OR "covid-19 sequelae"
