# Supplementary material for: Effects of Telerehabilitation on Quality of Life, Physical Capacity, and Symptom Burden Within the Post–COVID-19 Condition: Systematic Review and Meta-Analysis of Randomized Controlled Trials
Source: JMIR Rehabil Assist Technol. 2026 Oct 27;13:e95368. doi: 10.2196/95368 (PMC13752595; doi:10.2196/95368)
Supplement: Supplementary file 4 [file rehab_v13i1e95368_app3.docx]

**Multimedia Appendix 3.** Outcome measures, instruments, scoring direction, and orientation in the meta-analysis.

| Outcome | Instrument | Higher score indicates | Orientation for pooling | Data handling in meta-analysis |
| --- | --- | --- | --- | --- |
| HRQoL | SF-36 (physical component); | Better | As reported | SMD |
|  | SF-12; | Better | As reported |  |
|  | EQ-5D; | Better | As reported |  |
|  | WHOQOL-BREF; | Better | As reported |  |
|  | PROMIS preference (PROPr); | Better | As reported |  |
|  | K-BILD; | Better | As reported |  |
|  | SGRQ | Worse | Reversed |  |
| Aerobic capacity | 6MWT (metres); | Better | As reported | MD |
| Lower-limb function | 30-s / 1-min STS (repetitions) | Better | As reported | SMD |
|  | 5-repetition STS seconds; | Worse | Reversed |  |
| Dyspnea | mMRC; | Worse | Reversed | SMD |
|  | Multidimensional Dyspnea-12; | Worse | Reversed |  |
|  | BDI; | Worse | Reversed |  |
|  | TDI; | Better | As reported |  |
| Fatigue | Chalder FS / FAS / FSS | Worse | Reversed | SMD |
| Anxiety and depression symptoms | HADS (total or anxiety subscale); GAD-7 | Worse | Reversed | SMD |

“As reported” = entered without sign change; “Reversed” = the sign of the effect was multiplied by −1 so that a positive estimate consistently denotes benefit. Study names in parentheses indicate the trials contributing that instrument. BDI: Barthel Dyspnea Index; 6MWT: 6-minute walk test; EQ-5D: EuroQol 5-Dimension; GAD-7: 7-item Generalized Anxiety Disorder scale; HADS: Hospital Anxiety and Depression Scale; HRQoL: health-related quality of life; K-BILD: King’s Brief Interstitial Lung Disease questionnaire; MD: mean difference; mMRC: modified Medical Research Council scale; PROMIS: Patient-Reported Outcomes Measurement Information System; PROPr: PROMIS preference score; SF-12: 12-Item Short Form; SF-36: 36-Item Short Form Health Survey; SGRQ: St George’s Respiratory Questionnaire; SMD: standardized mean difference; STS: sit-to-stand test; BDI: Transition Dyspnoea Index.
