# Supplementary material for: Effects of Telerehabilitation on Quality of Life, Physical Capacity, and Symptom Burden Within the Post–COVID-19 Condition: Systematic Review and Meta-Analysis of Randomized Controlled Trials
Source: JMIR Rehabil Assist Technol. 2026 Oct 27;13:e95368. doi: 10.2196/95368 (PMC13752595; doi:10.2196/95368)
Supplement: Supplementary file 5 [file rehab_v13i1e95368_app4.docx]

**Figure 1.** Leave-One-Out Meta-Analysis for Health-related quality of life

**Figure 2.** Leave-One-Out Meta-Analysis for 6-Minute Walk Test

**Figure 3.** Leave-One-Out Meta-Analysis for Sit-to-Stand test

**Figure 4.** Leave-One-Out Meta-Analysis for Fatigue

**Figure 5.** Leave-One-Out Meta-Analysis for Hospital Anxiety and Depression Scale
